# Parasitism in Children Aged Three Years and Under: Relationship between Infection and Growth in Rural Coastal Kenya

**DOI:** 10.1371/journal.pntd.0003721

**Published:** 2015-05-21

**Authors:** A. Desiree LaBeaud, Monica Nayakwadi Singer, Maxim McKibben, Peter Mungai, Eric M. Muchiri, Elisabeth McKibben, Ginny Gildengorin, Laura J. Sutherland, Charles H. King, Christopher L. King, Indu Malhotra

**Affiliations:** 1 Division of Pediatric Infectious Diseases, Stanford School of Medicine, Palo Alto, California, United States of America; 2 Center for Immunobiology and Vaccine Development, UCSF Benioff Children’s Hospital Oakland, Children’s Hospital Oakland Research Institute, Oakland, California, United States of America; 3 Center for Global Health and Diseases, Case Western Reserve University, Cleveland, Ohio, United States of America; 4 Division of Vector Borne and Neglected Tropical Diseases, Ministry of Public Health and Sanitation, Nairobi, Kenya; University of Kelaniya, SRI LANKA

## Abstract

**Background:**

Parasitic infections, which are among the most common infections worldwide, disproportionately affect children; however, little is known about the impact of parasitic disease on growth in very early childhood. Our objective was to document the prevalence of parasitic infections and examine their association with growth during the first three years of life among children in coastal Kenya.

**Methodology/Principal Findings:**

Children enrolled in a maternal-child cohort were tested for soil transmitted helminths (STHs: *Ascaris*, *Trichuris*, hookworm, *Strongyloides*), protozoa (malaria, *Entamoeba histolytica* and *Giardia lamblia*), filaria, and *Schistosoma* infection every six months from birth until age three years. Anthropometrics were measured at each visit. We used generalized estimating equation (GEE) models to examine the relationship between parasitic infections experienced in the first three years of life and growth outcomes (weight, length and head circumference). Of 545 children, STHs were the most common infection with 106 infections (19%) by age three years. Malaria followed in period prevalence with 68 infections (12%) by three years of age. Filaria and *Schistosoma* infection occurred in 26 (4.8%) and 16 (2.9%) children, respectively. Seven percent were infected with multiple parasites by three years of age. Each infection type (when all STHs were combined) was documented by six months of age. Decreases in growth of weight, length and head circumference during the first 36 months of life were associated with hookworm, *Ascaris*, *E*. *histolytica*, malaria and *Schistosoma* infection. In a subset analysis of 180 children who followed up at every visit through 24 months, infection with any parasite was associated with decelerations in weight, length and head circumference growth velocity. Multiple infections were associated with greater impairment of linear growth.

**Conclusions/Significance:**

Our results demonstrate an under-recognized burden of parasitism in the first three years of childhood in rural Kenya. Parasitic infection and polyparasitism were common, and were associated with a range of significant growth impairment in terms of weight, length and/or head circumference.

## Author Summary

Parasitic infections are extremely common worldwide and children are especially vulnerable to these infections during critical periods of growth and development. There is a paucity of information about how frequently very young children (under the age of five years) are infected with parasites and the effects of parasitic infections on their growth and development. The findings from this study reveal that not only does infection occur early in life; it is associated with decreases in physical growth, even with low overall prevalence for some parasites. Decreases in growth of weight, length and head circumference during the first 36 months of life were associated with hookworm, *Ascaris*, *E*. *histolytica*, malaria and *Schistosoma* infection. In a subset analysis of 180 children who followed up at every visit through 24 months, infection with any parasite was associated with decelerations in weight, length and head circumference growth velocity. Multiple infections were associated with greater impairment of linear growth. It seems worthwhile to pursue a better understanding of prevalence and effects of parasitic infection in this vulnerable group to effectively target therapeutic interventions. And finally, if parasite transmission is to be significantly disrupted, control programs targeting these young, usually asymptomatic, age groups may be critical.

## Introduction

Parasitic infections, which are among the most common infections worldwide, disproportionately affect children [1]. It is increasingly recognized that both protozoan and helminthic diseases are common among children under the age of five years. Of particular concern, their associated disease burden is experienced during the period of life most critical for physical and cognitive development [1].

The association between parasitic infection and growth impairment has long been established [2]. However, few studies have evaluated the impact of parasitic disease on growth in very early childhood [3–5]. The mechanism by which parasitic disease impairs child growth is not fully understood, but is thought to be related to host systemic responses to infection (fever, decreased appetite), to intestinal disease disruption of host gut processes, and to anemia [2,4]. *Schistosoma japonicum* and *S*. *mansoni* infections have been associated with growth inhibition in school age children, even among those with low parasitic burden [2,6]. It is also evident that parasite-related growth deficits can be overcome by specific treatment of these infections. In a pediatric population in Kenya, weight gain deficits were ameliorated with intermittent albendazole treatment for soil transmitted helminth infections [7]. Other studies have shown improvement in growth and physical activity in children actively treated for STH infections [1,8].

Similar findings were seen in coastal Kenyan pre-school aged children infected with helminths [3]. In Peruvian pre-school aged children, Gyorkos and colleagues found a greater degree of stunting and reduced length-for-age Z-score in children infected with moderate to heavy helminth infections as compared to children with no or light infections [4]. However, such studies have not provided long-term longitudinal data on infants followed from birth.

The goal of the present prospective study was to monitor the incidence of childhood parasitic infections (intestinal parasites, malaria, filaria and *Schistosoma haematobium* infection) from birth to the age of 36 months in an area known to be endemic for multiple parasite species. This, in turn, provided better definition of the association between incident parasitic infection and growth impairment experienced during early childhood.

## Methods

### Ethics Statement

Healthy pregnant women and their offspring born at the Msambweni District Hospital on the south coast of Kenya were enrolled in this mother-child cohort study. Approval for the study was obtained from the Kenya Medical Research Institute National Ethical Review Committee and from the Institutional Review Board for Human Studies at University Hospitals of Cleveland Case Medical Center. Mothers provided written informed consent for their own participation and that of their infants.

### Study Design and Participants

This observational study was performed at Msambweni District Hospital on the southern coast of Kenya from 2007–2010. Healthy pregnant women and their offspring were enrolled in a three-year longitudinal maternal-child cohort study. Pregnant mothers were included if they were permanent residents of Msambweni and received pre-natal and post-natal care at the District Hospital’s antenatal clinic (ANC). Mother-infant pairs were excluded if: a) they experienced a complicated infant delivery resulting in significant infant morbidity at birth; b) the infant was born at less than 36 weeks gestation; c) the mother had known chronic illness; d) the mother had severe anemia (Hgb < 6 g/dl) requiring hospitalization and urgent treatment; e) the mother had permanent disability that impeded study participation and/or comprehension; or f) the family had plans to relocate after delivery.

Upon enrollment, mothers completed a detailed questionnaire that queried their education level, spouse’s occupation, and household income. At later time points, they and their offspring were screened for malaria, *Schistosoma haematobium*, filariasis, and intestinal helminthes and protozoa. Parasitic infections were tested via blood, urine and stool. Sera and plasma were frozen at -20°C for subsequent determination of filarial Og4C3 antigen testing, filarial BMA specific (*Brugia malayi* antigen) IgG4 antibody, and *Schistosoma haematobium* specific (soluble worm antigen of *S*. *haematobium*) IgG4 levels. Red blood cell (RBC) pellet was used for DNA extraction for detection of malaria parasites via RTQ-PCR [5]. A drop of blood was used to make a malaria blood smear and measure hemoglobin by Hemocue method [9,10]. Malaria positive was defined as either positive blood smear or positive PCR or both. *W*. *bancrofti* infection was detected by assay for circulating antigen in plasma samples with the use of a commercial Og4C3 antigen detection assay (TropBioMed, Townsville, Australia) and also assessed by ELISA detection of BMA-specific IgG4 antibodies [11–13]. Positive filaria infection was defined as either positive Og4C3 antigen or presence of BMA-specific IgG4 antibodies or both. A single sample of fresh stool was examined in duplicate to quantify ova of intestinal parasites using the Ritchie Method [14]. A single fresh urine sample was filtered and examined for *S*. *haematobium* eggs [11,13]. Infection status with *S*. *haematobium* was also assessed by ELISA detection of SWAP-specific (soluble worm antigen of *S*. *haematobium*) IgG4 antibodies in plasma samples [13]. Positive *S*. *haematobium* infection was defined as positive for either eggs identified in urine or the presence of SWAP-specific IgG4 antibodies or both.

Infants were followed from birth every six months until three years of age. Twins were included and analyzed as individuals since they comprised <2% of the cohort. In addition to follow up visits, children were evaluated during any episodes of acute illness that occurred between scheduled visits. A select group of specially-trained clinical staff performed physical examinations, including anthropometrics (weight, length and head circumference) on all infants at delivery and at each scheduled follow up visit. Following Kenya Ministry of Health guidelines, infants received amodiaquine (if <5 kg) or artemether/lumefantrine (Coartem) (if >5 kg) for malaria treatment. Infants were treated with mebendazole for soil transmitted helminth infections.

### Statistical Analysis

For purposes of analysis, we first transformed the growth outcomes into age and gender-specific Z-scores, using the WHO Anthro software (WHO, Geneva, Switzerland) [15]. The Z-score is a standardized score indicating how many standard deviations a child’s growth parameter differs from an established mean value for children of the same age among the world’s population. For our analysis, the key dependent variables of interest were weight, height and head circumference Z-scores over the first three years of life. The main exposure variables of interest were the number and types of infection at any time point (cumulative infections in mothers and their offspring). Basic descriptive analyses of all the measurements were conducted on each time point in the study. The statistical analysis was performed using SAS version 9.3 (SAS Institute Inc., Cary NC).

In initial exploratory analysis for bivariate comparisons of continuous data, the Student’s t-test was used, and for categorical data, chi-square tests were used. Next, logistic regression models were created to assess the association of maternal infection during pregnancy with infant infection at each follow up visit. The primary study outcome, the association of infant infection at any point prior to the measured time point (cumulative infections) with the rate of growth and growth at each time point, was evaluated using general estimating equations models (GEE) for longitudinal data. The use of GEE models allowed us to account for the heteroscedasticity of repeated outcome measurements on the same subjects over time. For soil transmitted helminth infections, infection by each species was analyzed alone (*Ascaris*, *Trichuris*, hookworm, or *Strongyloides*) and then as an aggregate class (“STH”). Infections were treated as categorical, either present or absent for all types. The final models examined the relationship between the infant’s parasitic infections and their weight, length, and head circumference Z-scores over the first three years of life, while controlling for the infant’s sex, birth weight, birth length, birth head circumference, and maternal education. Because maternal education was highly correlated with household expenditures, it was used as a proxy for socioeconomic status. To control for secular changes during the study, infant’s infection, time, and the interaction of infection and time were included in the models. Tukey’s method of adjustment was used to assess significance when comparing across multiple time points. A significance level of 0.05 was used for all statistical tests.

To better refine the estimates of the effects of incident infection on growth trajectory, a subset analysis was performed on the cohort of 180 children who did not have any missed scheduled visits from birth to 24 months. This 24 month endpoint was chosen for this analysis because of a significant limitation in the number of children who were able to complete every visit through 36 months (only 91 children).

## Results

### Infection Prevalence

During the study period, 545 infants were followed and tested for parasitic infections over the first three years of life. Of the 545, 13 pairs of twins (26 infants) were included. Maternal and infant characteristics are described in Table 1.

**Table 1:** Maternal and infant characteristics.

Characteristic	
**Maternal (N = 545)**	
**Maternal Age at Delivery (years)**	
14–22	153 (28%)
23–30	210 (39%)
>30	87 (16%)
Unknown	95 (17%)
**Education**	
None	100 (18%)
Lower Primary	94 (17%)
Upper Primary	263 (48%)
Secondary or more	86 (16%)
Unknown	2 (1%)
**Household Income** (KSh* per month)	
<3000	322 (59%)
>3000	221 (40%)
Unknown	2 (1%)
**Infant at Delivery (N = 545)**	
Male	288 (55%)
Female	236 (45%)
Average Head Circumference (cm^**€**^)	34.2 +/- 1.4 (range: 27.5–38)
Average Length (cm)	48.6 +/- 2.5 (range: 40–59.5)
Average Weight (g^**¥**^)	2949 +/- 492 (range: 1100–4350)

*KSh: Kenyan Shillings

^**€**^cm: centimeters

^¥^g: grams

**Fig 1:**
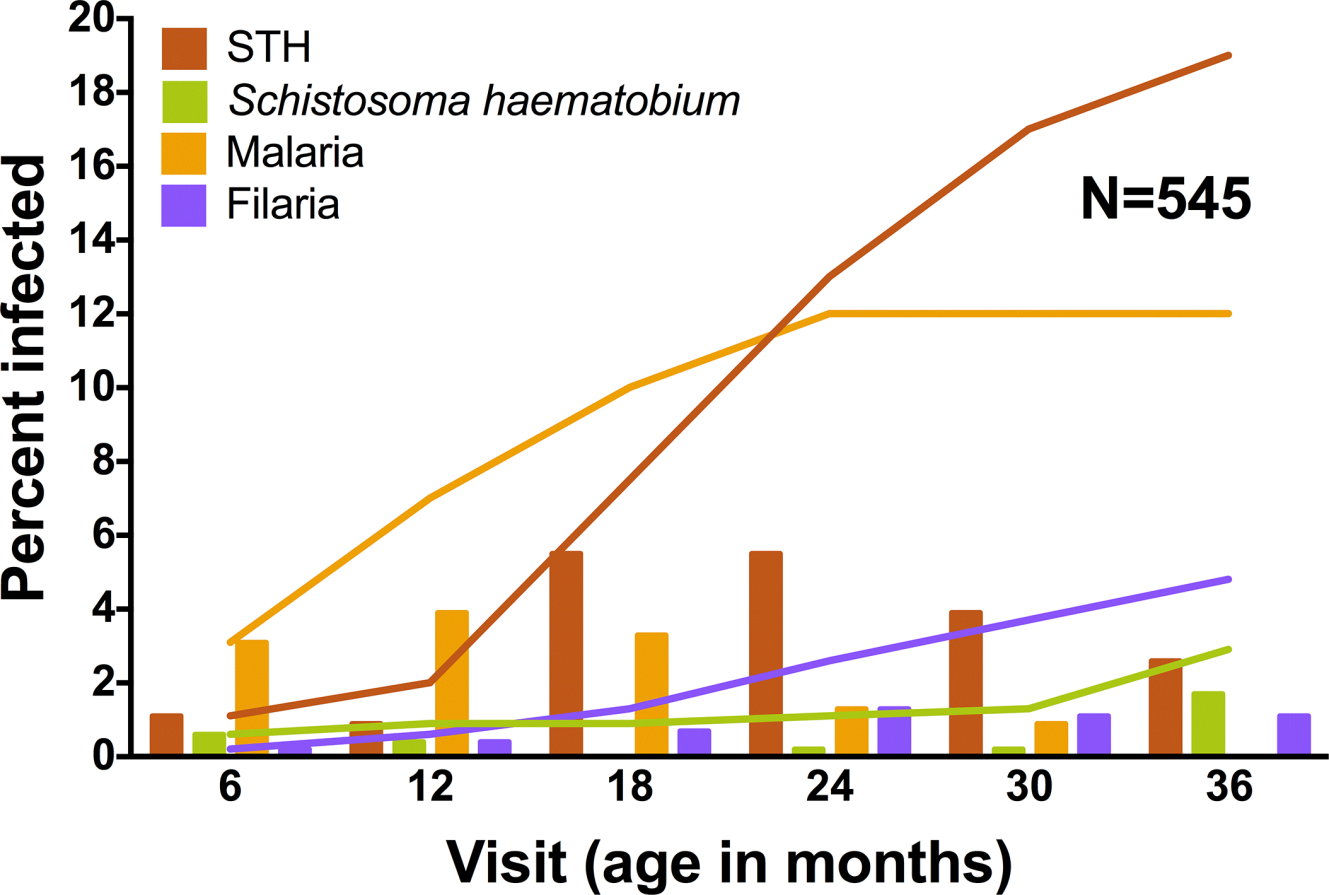
Prevalence of infection at regular study follow-up visits from 6–36 months of age. Bars represent the percent of children infected at each visit. Lines represent cumulative incidence of infection over time.

**Fig 2:**
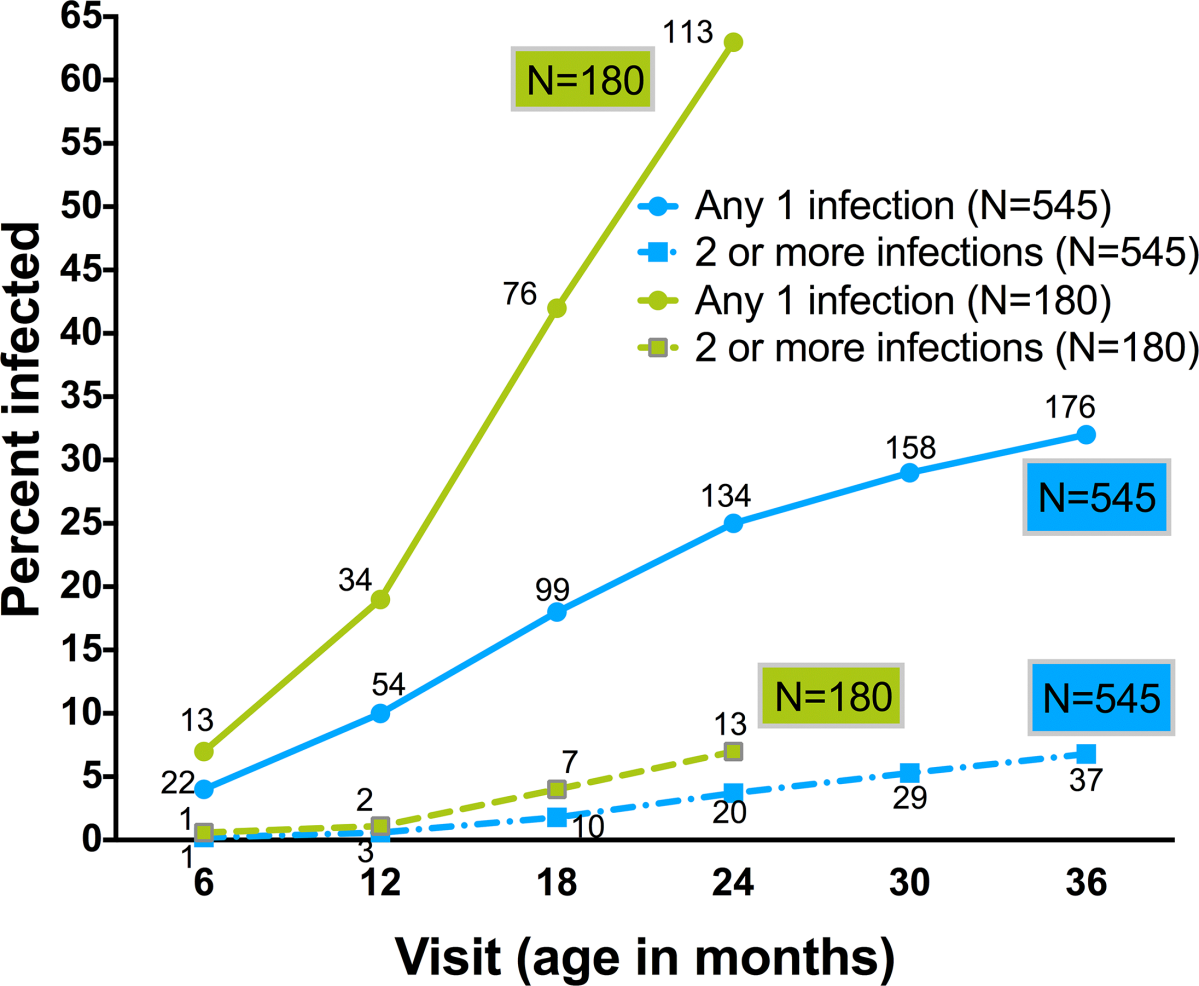
Cumulative incidence of parasitic infections. Shown are rates of any parasitic infection (solid lines with circles) or of >1 (multiple) parasitic infections (dashed lines with squares) by the age of each study visit during the first 36 months of life for the total study cohort (N = 545), and for the first 24 months of life for the full follow-up cohort (N = 180). The number of children infected at each time point is indicated near the marker.

During the infant observation period, soil transmitted helminths were the most common infection, with 106 of 545 or 19% (CI_95_: 16–23%), of children experiencing infections by age three (Fig 1). Malaria followed in period prevalence with 68 (12%, CI_95_: 10–16%) infected by three years of age. Filaria and *S*. *haematobium* infection occurred in 26 (4.8%, CI_95_: 3–7%) and 16 (2.9%, CI_95_: 2–5%) children, respectively. Thirty-two percent (176 of 545) of children experienced at least one parasitic infection and 7% (37 of 545) were infected with more than one parasite in the first 36 months of life (Fig 2). All infection categories were documented as early as six months of age. For *Schistosoma* infection, the infants less than 18 months were only positive by IgG4 assays. Of the three positive infants positive for filarial infection under 18 months of age, one was only positive via BMA IgG4 assay. Prenatal maternal infections increased the odds of infant infections (see S1 Table).

### Association of Infection with Changes in Growth Parameters

**Fig 3:**
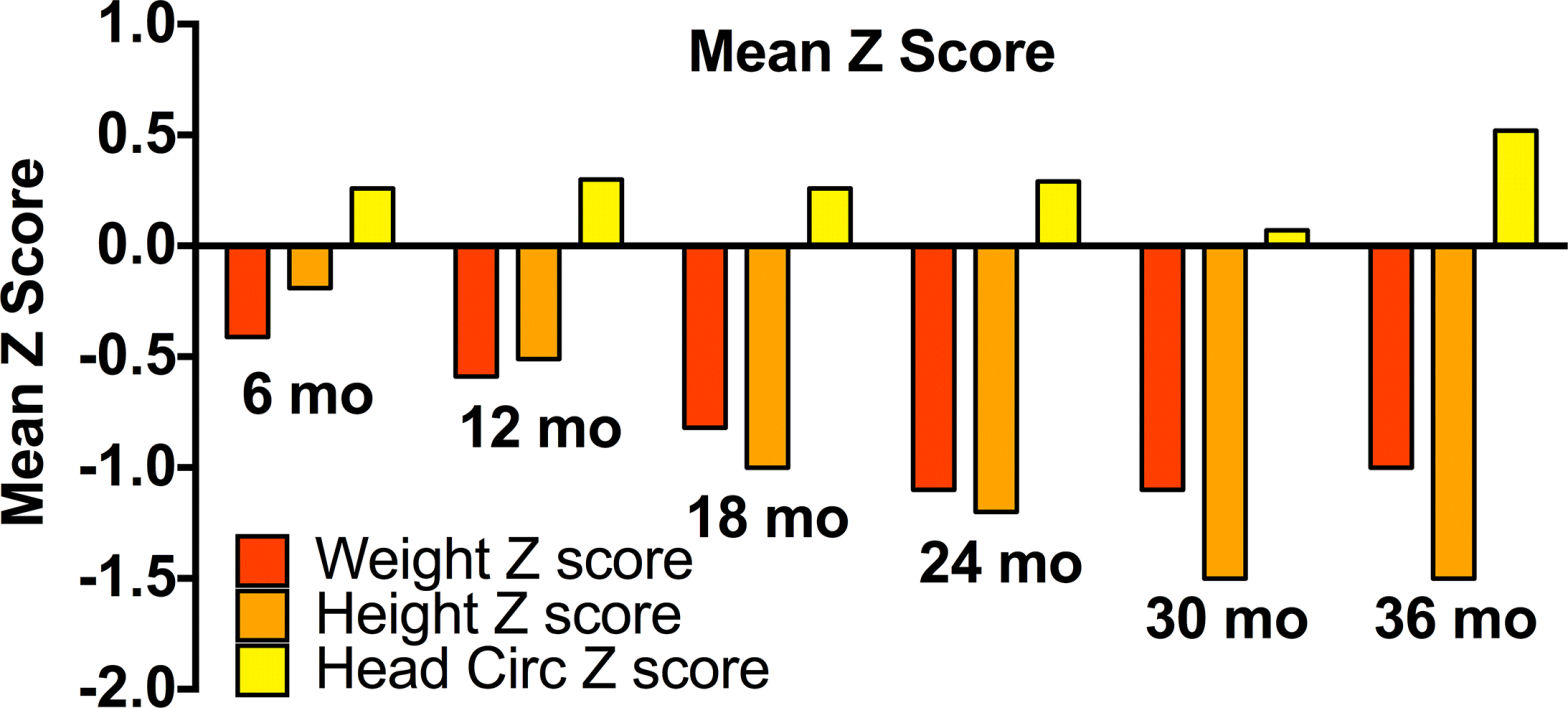
Average length, weight and head circumference Z-scores. Data summarized for all children (N = 545) at each follow up time point.

Infant parasite infection in the first three years of life was associated with decreases in several growth parameters (Table 2), although prevalence of marked stunting and wasting were low overall (<2% of cohort for each). The average length, weight and head circumference Z-scores for all children at each follow up time point are shown in Fig 3. STH infection by 24 months was significantly associated with a lower growth attainment in terms of length and head circumference relative to WHO standards. STH infection by 30 months was also associated with a lower growth in head circumference by 30 months.

**Table 2:** Results of longitudinal models describing the association between infant parasitic infection and growth parameters.

Infection		N = 545 Cohort^a^				N = 180 Cohort^b^		
Hookworm		Weight		Length	Head Circumference	Weight	Length	Head Circumference
	Age (mo)							
	24	ns^c^		↓-0.36, p = 0.001^d^	↓-0.66, p = 0.002	↓-0.53, p = 0.02	ns	↓-0.62, p = 0.02
	30	ns		ns	↓-0.73, p = 0.004	ns	ns	ns
	36	↓-0.43, p = 0.05		ns	ns	ns	ns	ns
***Trichuris***	6	ns		ns	ns	↓-0.47, p = 0.04	ns	ns
***Ascaris***	6	↓-0.61, p = 0.02		ns	ns	ns	ns	ns
	12	↓-0.66, p = 0.01		ns	ns	ns	ns	ns
	18	↓-0.58, p = 0.03		ns	ns	ns	ns	ns
	24	ns		↓-0.93, p<0.001	ns	ns	↓-0.86, p = 0.02	ns
	36	ns		ns	↑+1.39, p = 0.007	ns	ns	ns
***E*. *histolytica***	24	↓-0.37, p = 0.03		ns	ns	ns	↓-0.51, p = 0.04	ns
	30	ns		ns	↓-0.51, p = 0.04	ns	ns	ns
***Giardia***	12	ns		ns	ns	ns	↓-0.50, p = 0.03	ns
***Strongyloides***	6	ns	ns		ns	↑+1.17,p = 0.04	ns	ns
	24	ns	ns		ns	↑+1.22,p = 0.03	ns	ns
	30	ns	ns		↓-1.69, p = 0.002	ns	ns	ns
**STH**	24	ns	↓-0.35, p = 0.01		↓-0.47, p = 0.007	ns	ns	ns
	30	ns	ns		↓-0.41, p = 0.03	ns	ns	ns
***S*. *haematobium***	24	ns	ns		ns	ns	↑+0.8, p = 0.05	ns
	36	ns	ns		↓-1.07, p = 0.02	ns	ns	ns
**Malaria**	6	ns	↓-0.81, p = 0.002		ns	ns	ns	ns
	12	ns	↓-0.41, p = 0.02		ns	ns	ns	ns
	18	ns	↓-0.46, p = 0.003		ns	↓-0.35, p = 0.05	↓-0.64, p = 0.003	
	24	ns	ns		↓-0.66, p<0.001	ns	ns	↓-0.77, p<0.001
	36	ns	ns		↓-0.62, p = 0.01	ns	ns	ns
**Filaria**	24	ns	ns		ns	↑+0.73, p = 0.03	ns	ns

Shown are the multiply-adjusted impacts of infection on weight, length and head circumference Z-scores at follow up age milestones.*

*Longitudinal Models: Controlled for age, sex, birth weight, birth length, birth head circumference, and maternal education.

^a^N of 545 cohort represents all of the children followed over the first 36 months of life.

^b^N of 180 is a subset of the 545 cohort representing the children following up at every visit in the first 24 months of life.

^c^ns: not statistically significant.

^d^Tukey adjusted p-value with the effect size (+/-) and arrow indicating direction of effect.

When evaluating soil-transmitted helminths individually, hookworm infection by 24 months was associated with below average growth in length and head circumference at 24 months and relative deficit in overall weight gain over the first 36 months of life. Hookworm infection by 30 months was associated with a decreased Z-score for head circumference at 30 months, while infection by 36 months of age was associated with a relative decrease in weight gained at 36 months. *Ascaris* infection by either 12 or 18 months was associated with a decrease in weight Z-scores at each age milestone, respectively. *Ascaris* infection by 24 months was associated with a decrease in length achieved at 24 months. *Strongyloides* infection by 30 months was associated with a relatively lower head circumference at that age.

Of intestinal protozoa, *Entaomeba histolytica* infection by 24 months was associated with a lower weight Z-score at 24 months, and infection by 30 months was associated with a lower head circumference score at 30 months.

*Schistosoma* infection by 36 months was associated with a reduced Z-score for head circumference at 36 months. Malaria infection was associated with a lower linear growth (length) attainment at 6, 12 and 18 months and lower Z-scores for head circumference at 24 and 36 months. Of note, in the study cohort, no significant reductions were observed in terms of weight, length, or head circumference scores following filarial infection.

In the subset of 180 infants who attended all follow up visits through two years of age, as in the overall group (N = 545), soil transmitted helminths proved to be the most common infection (47 infants, 26%, CI_95_: 19–31%) by 24 months of age.

Among this more closely followed group of children, a number of growth deficits were significantly associated with infection. Hookworm infection by 24 months was associated with a decrease in weight and head circumference Z-score at 24 months (Table 2). *Ascaris* infection by 24 months was associated with a decrease in linear growth (length Z-score) at 24 months. *E*. *histolytica* infection by 24 months was also associated with a length deficit by 24 months. Malaria infection was associated with a statistically significant reduction in weight and length attained at 18 months, and in head circumference at 24 months. By contrast, filarial infection was associated with a statistically significant increase in weight attained at 24 months. Among this full follow-up group, the relative rate of growth attained in weight, length or head circumference by 24 months was decreased among children with any parasitic infection as compared to the uninfected group (Table 3).

**Table 3:** Relative change in Z-score in children infected with any parasite by 24 months of age.

Growth Parameter	Direction of effect (Size)	P value^a^
WEIGHT	Decreased (-2.28)	0.021
LENGTH	Decreased (-0.33)	0.022
HEAD CIRCUMFERENCE	Decreased (-2.61)	0.009

Multiply-adjusted impact based on **l**ongitudinal models that controlled for age, sex, birth weight, birth length, birth head circumference, and maternal education (N = 180).

^a^Tukey adjusted p-value of the effect size.

Between the group with complete follow up and the cohort with incomplete follow up (*i*.*e*. N = 545 cohort minus 180), there were significant differences between maternal education and number of infections. In the complete follow up group (N = 545), mothers had higher education (p = 0.005) and children experienced more infections (p = 0.004) over the first 24 months of life.

### Polyparasitism Effects

**Fig 4:**
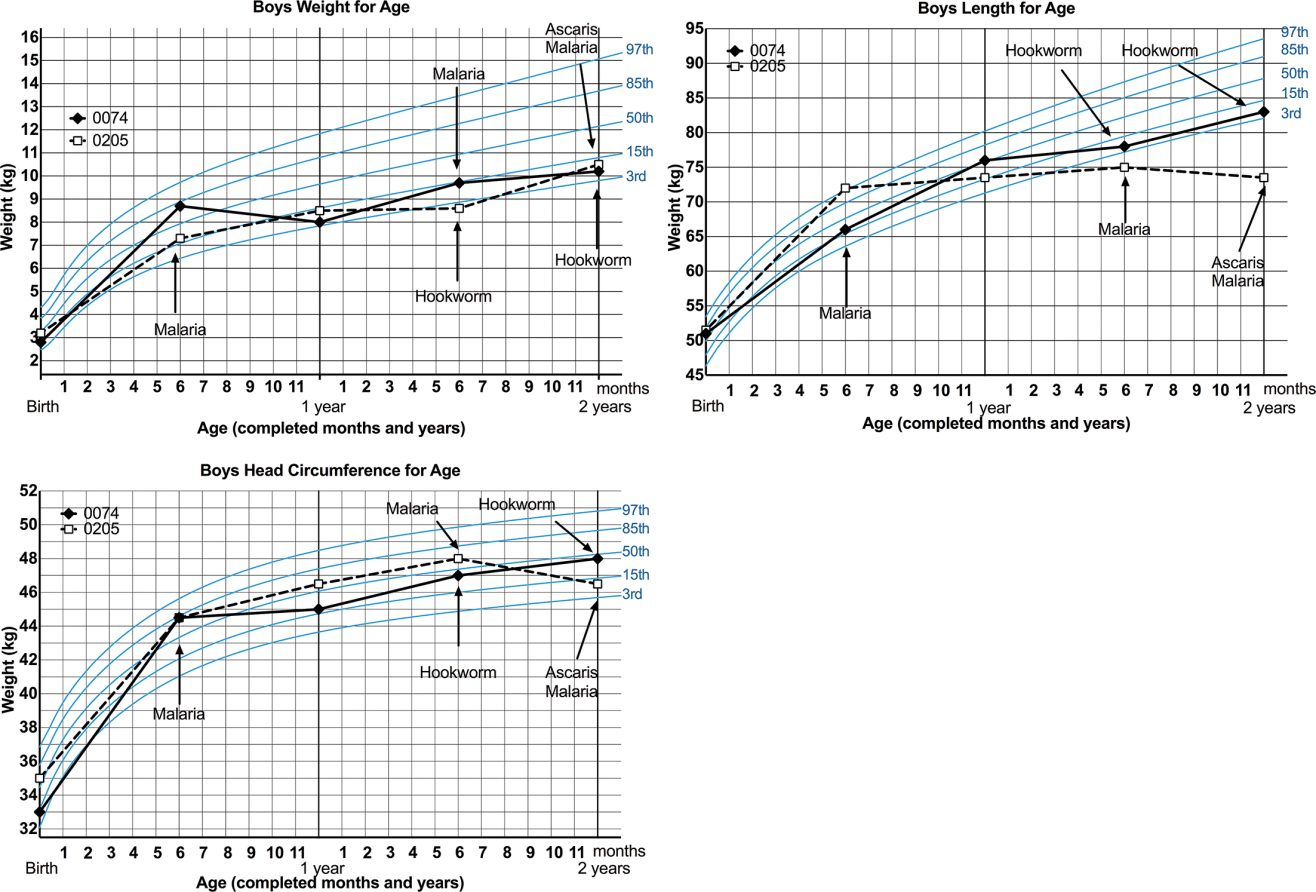
Impact of multiple parasitic infections on growth. WHO-standardized weight, length and head circumference growth charts for two male infants experiencing multiple parasitic infections in the first 24 months of life.

Overall, polyparasitism (defined as children experiencing more than one type (any soil transmitted helminth, filaria, malaria and/or *Schistosoma haematobium*) of parasitic infection at any time point) was associated with decreases in length (p = 0.004) by 36 months of life as compared to children who did not experience any parasitic infections in the first 36 months of life. To demonstrate the relative timing of polyparasitism effects on infant growth, WHO growth charts are shown for two infants who experienced multiple parasitic infections in the first 24 months of life (Fig 4).

## Discussion

Our results confirm that parasitic infections are common among the youngest age groups of children (under three years old) who live in endemic areas such as rural coastal Kenya. This study found that parasitic infections during the first three years of life were associated with significant differences in growth and that children can be infected within the first six months of life. By 24 and 36 months of age, many children were multiply infected (polyparasitized), with 7% of the children experiencing more than two different parasitic infections.

STHs were the most common infections encountered in our cohort. This is not surprising as young children have frequent contact with contaminated soil and explore their environment by touching and tasting early in life. An increase in the absolute number of STH infections was observed between six to 18 months, at the time when children are becoming mobile. STHs infect the gut, a compartment with relatively limited effects of host immunity, free from the robust response of the immune system found in host tissues and circulation, and have a constant supply of nutrients. They can successfully inhabit this environment for long periods of time, resulting in prolonged chronic infection [16]. Intestinal worms may disrupt nutrition by 1) feeding on host tissue causing blood, iron, and protein loss; 2) impairing digestion and absorption; 3) causing generalized inflammatory responses that lead to decreased appetite and increased metabolic demand, thus, diverting nutrients and energy of the host [1,2,16]. Hookworms are one of the most important species in terms of disease causing profound anemia even at low egg counts [1]. *Ascaris* and hookworm were associated with decreased growth in terms of weight, body length, and head circumference, likely via the mechanisms mentioned earlier.

In addition to intestinal parasitic diseases, other parasites such as malaria and *Schistosoma* infection often lead to anemia and contingent host inflammatory responses, resulting in decreased energy intake and compromised host nutrition [6,16]. In particular, malaria was associated with multiple growth deficits in our cohort. Overall, our data indicate that growth effects were more often significant at 24 and 36 months of age, likely because the deleterious effects of infection take time to significantly retard growth. Lower height-for-age Z-scores (i.e. stunting) indicates long term insult (chronicity), whereas, lower weight-for-length Z-score represents acute insult. In a resource-limited setting, these deficits likely represent both the effects of parasitic infection and the effects of comorbidities such as undernutrition. It is important to note how early in life these effects can be detected.

*Schistosoma* infection was associated with a decrease in head circumference Z-score at 36 months; a finding that was statistically significant, even though only a relatively small number of children were infected. However, it is worth noting that these young children were only positive for *Schistosoma* infection via antibody testing, which could be the result of persistent maternal IgG4 antibody in the child’s circulation. Whatever the source of the positive serology, it is worth noting that this group of children experienced alteration in growth either from exposure to *Schistosoma haematobium* in utero, or from early life infection. Malaria, hookworm, and other soil transmitted helminth infections were also associated with decreases in head circumference. The rate of head growth during the first three years of life is significantly correlated with cognitive development and brain growth [2,17], and it is notable that, significant cognitive developmental delays have been associated with other types of infection experienced in early life [5,16,18]. Future directions for study should include evaluation of the associations between parasitic infections, decreased growth in head circumference, and loss of cognitive potential, especially in the first two years of life, as this is the period known to be most important for brain development.

Over the three year period of the study, follow up of the 545 children was incomplete, such that inclusion of different children in different age milestone data groups likely led to variability in the strength of associations between infections and growth outcomes. To overcome this limitation, we performed a subgroup analysis of children who were followed at every visit until the age of two. Among these children, cumulative infection rates were higher, likely due to more frequent testing and better ascertainment of early infection status. Furthermore, among this group, larger growth rate deficits were documented in weight, length and head circumference in infected children. The relative rate of change in Z-scores for weight, length and head circumference over the first 24 months of life (i.e. growth velocity) was decreased in children with any parasitic infection versus children without infection.

Unexpectedly, we observed some associations between parasitic infections and an increase in growth parameters. Filaria infection by 24 months was associated with an increase in weight Z-score. Studies in experimental animal models have shown an association between filarial infection and obesity in mouse models [19]. Our cohort children, although they had no clinical signs of lymphedema, may have had excess inflammation with interstitial fluid and fat deposition, which might explain this observation. *Ascaris* infection by 36 months of life was associated with a significant increase in head circumference Z-score. Again, we cannot currently explain this finding based on individual child, family, or SES factors.

Polyparasitism, which was defined as infection with more than one parasite at any given time during the first 36 months of life, was associated with growth faltering in a dose dependent relationship. The number of child infections over the three years was found to be negatively associated with length changes over time. While most previous studies have correlated poor growth outcomes with intensity of infection, the finding of multiple infections associated with linear growth in early life is novel [4].

An interesting finding with respect to estimated socio-economic standing (SES) was the better rates of follow up among the lower SES families. This may have been due to a greater number of working women in the higher SES groups, making adherence with regular follow up visits more challenging.

There were strengths and limitations for this study. We recognize that a cohort bias exists, as we were comparing infants within one district over a limited period of time, and did not evaluate growth patterns or parasitic prevalence outside this community. We acknowledge that there are many other factors that may have contributed to poor growth and nutrition in this resource limited setting, including lack of access to protein-rich foods, and intercurrent illness with diarrhea or other conditions. Although we have data on the children’s care while ill between visits, we did not have data on other potential co-infections or comorbidities that might have impacted growth. Unlike some previous studies, we did not attempt to tease out details of nutritional status, factors that could have had confounding effects on growth parameters related to chronic anemia, iron and micronutrient deficiencies, and lack of calorie-rich foods. Use of comprehensive 24 hour food recall questionnaires and iron or micronutrient serum markers would have bolstered our anthropometric assessments. However, weight, length and head circumference for age and sex are, in and of themselves, very reliable tools for growth assessment when performed by trained teams, as in our study [15,20,21]. As for any tool that requires human measurement, both random and systematic error can be introduced into the measured data. Inter-rater variability was reduced in this study by using the same staff, who were fully trained to perform standardized measurements, throughout the study period. Because we had precise knowledge of each child’s birth date, and, hence, their age in days, our Z-scores were likely to be more accurate than those reported in past cross-sectional surveys, where children’s ages have based on recall, which is much less reliable. In our study, food availability was not specifically measured, however, maternal education, used as a proxy for socioeconomic standing, was used to estimate access to nutrition. In terms of other environmental factors, we could not measure the complex interaction of other risk factors for acquiring infection, *i*.*e*., lack of shoes increasing risk of hookworm infection and poor sanitation, which cannot be simply derived from SES parameters and infection status. The longitudinal analysis of the association of infection with growth parameters required the use of cumulative infection effects instead of those of current infections at each time point. This was due to the small number of infected children at any given time point. Further, although the children were treated, we did not test children for cure, such that infections could have persisted. As a result, the cumulative effect of detected infections on growth may have been a more accurate reflection of the effects of infection in our cohort. We did not analyze the correlation between infection intensity and growth. Those infections with intensity data collected (schisto and STH) would have all been classified as “light” infections; therefore, all infections were categorized as either present or absent. Previous studies have found higher intensity STH infection to correlate with decreased body length in young children and likely an important contributor to overall poor nutrition and growth [4]. We recognize that many standard methods for testing for STH parasitic infections are not sensitive overall, and thus, infection rates we measured could have been falsely low, thereby leading to a Type II error [22,23]. By using multiple testing methods, sensitivity for detecting malaria, filarial and *Schistosoma* infections was increased in our study. However, testing with antibody assays for *S*. *haematobium* infection and filariasis in children less than 18 months may have been biased by persisting maternal antibody leading to misclassification bias (false positives). Despite these limitations, we feel that this longitudinal cohort study offers a more accurate documentation of the early onset parasitic infections among young children in the tropics and their effects on early growth.

Currently, most deworming campaigns are geared toward school-age children, among whom the impact of helminth infections has been well established. Operationally, this is the more “cost efficient” age group on which to focus therapy. However, this current ‘preventive chemotherapy’ policy is partly based on the notion that parasitic infection in the first five years of life is insignificant. Additionally, it is believed that deworming more heavily infected school-age children will yield a greater impact on parasite transmission community-wide. It is thus assumed that school age deworming will provide “trickle down” benefits in terms of growth and cognitive development to pre-school children. However, the findings of the present study reveal that helminthic parasite infection can occur very early in life and is associated with decreased physical growth, despite the low overall community prevalence for some of these parasites. It will be worth pursuing a better understanding of prevalence and effects of infection in these vulnerable pre-school age groups to most effectively target therapeutic interventions. Finally, if parasite transmission is to be fully disrupted, control programs must also logically target the younger, usually non-symptomatic age groups to prevent environmental contamination via egg dissemination.

New prospective cohort studies are now underway in our study area to evaluate the associations between parasitic infections and growth, development, physical fitness, quality of life and vaccine response in both preschool and school aged children. These studies will address many of the limitations discussed here.

## Supporting Information

S1 Checklist STROBE checklist. (DOC)

S1 Dataset De-identified dataset used for all analyses presented in this manuscript. (XLS)

S1 Table Odds ratios of infant parasitic infection at each follow up visit with respect to maternal prenatal parasitic infections. (DOCX)
