# Supplementary material for: Parasitism in Children Aged Three Years and Under: Relationship between Infection and Growth in Rural Coastal Kenya
Source: PLoS Negl Trop Dis. 2015 May 21;9(5):e0003721. doi: 10.1371/journal.pntd.0003721 (PMC4440755; doi:10.1371/journal.pntd.0003721)
Supplement: S1 Table — (DOCX) [file pntd.0003721.s003.docx]

**SUPPLEMENTAL DATA: MATERNAL PRENATAL INFECTION INCREASED ODDS OF INFANT INFECTION**

Mothers who experienced any type of parasitic infection during pregnancy had infants who were two times more likely to have an infection with any type of parasite in the first 36 months of life (Table A1). Mothers with soil transmitted helminth infections during pregnancy were two times more likely to have infants infected with STHs at 24, 30 and 36 months of age. Prenatal maternal malaria infection was associated with a two times greater likelihood of infant infection at 30 and 36 months. Maternal infection of any type during pregnancy did not have an association with infant weight, height or head circumference at delivery.

**TABLE S1: Odds Ratios of infant parasitic infection at each follow up visit with respect to maternal prenatal parasitic infections.**

| **MATERNAL INFECTION** | **INFANT INFECTION** | **VISIT (MONTHS)** | **OR (95% CONFIDENCE INTERVAL)** | **P VALUE** |
| --- | --- | --- | --- | --- |
| STH | STH | 24 | 1.71 (1.03-2.84) | 0.039 |
|  | STH | 30 | 2.05 (1.30-3.23) | 0.002 |
|  | STH | 36 | 1.95 (1.26-3.00) | 0.003 |
| Malaria | Malaria | 30 | 2.02 (1.09-3.76) | 0.026 |
|  | Malaria | 36 | 2.02 (1.09-3.76) | 0.026 |
| At Least One Parasitic Infection | At Least One Parasitic Infection | Over First 36 Months of Life | 1.95 (1.35-2.82) | <0.001 |

Early childhood parasite infections were more likely when mothers had been diagnosed with infection during prenatal care. Pregnant mothers experiencing any parasitic infection, specifically STH infection or malaria, were more likely to have infants with the same category of infection. This is expected, as mothers and their infants have the same environmental exposures and likely share heritable risk factors. However, this relationship was significant only at later age timepoints (beyond 24 months). This could reflect the time required to have sufficient exposure to infection, possibly modified by the child being carried by the mother earlier in life and thus, not exposed to soil as frequently. Caregivers may also have been more vigilant about infection precautions in the earlier months of the child’s life (*e.g*., use of bednets and/or water treatment).
